# Lipidomic Profiling Reveals Stage-Associated Triglyceride Accumulation in Macrophages in a Mouse Model of Ovarian Cancer

**DOI:** 10.3390/cancers18182959

**Published:** 2026-09-14

**Authors:** Bhawna Deswal, Elahe Nikpayam, Dong-Joo Cheon

**Affiliations:** Department of Regenerative and Cancer Cell Biology, Albany Medical College, Albany, NY 12208, USA; deswalb@amc.edu (B.D.); nikpaye@amc.edu (E.N.)

**Keywords:** ovarian cancer, tumor-associated macrophages, lipid metabolism, lipidomics, triglycerides

## Abstract

**Background/Objectives**: Tumor-associated macrophages (TAMs) are critical mediators for immunosuppressive ovarian tumor microenvironments. However, the lipid metabolic features of macrophages at different stages of ovarian tumor development remain poorly understood. **Methods**: Here, we performed LC-MS-based lipidomic profiling of CD11b^+^/F4/80^+^ macrophages isolated from ID8 tumor-bearing C57/BL6 mice at Days 10, 40, and 80 following tumor cell inoculation. **Results**: Principal component analysis and hierarchical clustering revealed stage-associated differences in macrophage lipid profiles. Phosphatidylethanolamine (PE), phosphatidylserine (PS), and lysophosphatidylcholine (LPC) were relatively more abundant at earlier time points, whereas triglyceride (TG) species were more abundant in macrophages isolated at Day 80. Detailed analysis revealed an increased abundance of TG species with greater total numbers of double bonds at Day 80. Pathway analysis identified enrichment of glycerolipid metabolism at Day 80. Consistent with these lipidomic findings, macrophages at Day 80 exhibited increased expression of lipogenic regulators, including *Fasn*, *Pparg*, and *Srebp1*, together with a gene expression profile associated with a pro-tumoral macrophage state. Supplementation of oleic acid to macrophages increased expression of selected genes associated with this macrophage state, supporting a potential relationship between altered lipid metabolism and macrophage gene expression changes. **Conclusions**: These findings identify stage-associated TG accumulation and alterations in macrophage lipid metabolism and provide a basis for future studies to determine whether macrophage lipid metabolism contributes to a pro-tumoral macrophage state.

## Simple Summary

Ovarian cancer is associated with increased infiltration of tumor-associated macrophages, which has been linked to poor clinical outcomes. Metabolic alterations within the ovarian tumor microenvironment have been shown to promote tumor progression, but the lipid metabolic features of macrophages at different stages of ovarian tumor development remain elusive. In this exploratory study, we performed lipidomic profiling of CD11b^+^/F4/80^+^ macrophages isolated from C57BL/6 female mice at Days 10, 40, and 80 following intraperitoneal injection of ID8 mouse ovarian cancer cells. We observed stage-associated changes in macrophage lipid composition, characterized by a shift from membrane-associated lipid classes toward increased abundance of triglyceride species at the late tumor stage. These changes were accompanied by altered expression of genes involved in lipid synthesis and storage and a gene expression profile associated with a pro-tumoral macrophage state. Supplementation of exogenous fatty acids to macrophages increased expression of selected genes associated with this macrophage state, supporting a potential relationship between lipid accumulation and macrophage gene expression changes. Together, these findings identify stage-associated alterations in macrophage lipid metabolism in a mouse model of ovarian cancer and provide a basis for future studies investigating whether macrophage lipid metabolism contributes to a pro-tumoral macrophage state and tumor progression.

## 1. Introduction

Ovarian cancer remains one of the deadliest gynecological malignancies worldwide, largely due to late diagnosis, and recurrence due to the development of chemoresistance [1]. Despite advances in surgical and chemotherapeutic strategies, the overall survival rate for patients with advanced ovarian cancer remains poor, which is characterized by a highly immunosuppressive tumor microenvironment (TME) and distant metastases [2]. Within this immunosuppressive TME, tumor-associated macrophages (TAMs) have emerged as key orchestrators of disease progression, where they attain pro-tumoral phenotype and promote distant metastasis [3]. Accumulating evidence revealed that metabolic reprogramming of TAMs plays a crucial role in increased their tumor-promoting properties in different type of cancers, including ovarian cancer [4,5].

Lipids are essential for diverse biological functions, including membrane synthesis, energy storage, and signaling [6]. In macrophages, lipids regulate the inflammatory response, phagocytotic functions and immune regulation [7]. The ovarian cancer microenvironment is lipid-rich; thus, macrophages in ovarian cancer are metabolically rewired to survive under metabolic stress [7]. Alterations in fatty acid synthesis, lipid uptake, and lipid storage can thereby skew macrophage function to support rapid tumor cell proliferation [5]. Recent advances in Liquid Chromatography–Mass Spectrometry (LC-MS)-based lipidomic have enabled comprehensive identification and characterization of lipid species in biological systems, providing deeper insight into lipid metabolic alterations associated with disease [8]. Lipidomic analyses can determine significant changes in lipid classes in cells and tissues in tumor, including glycerophospholipids, sphingolipids, and glycerolipids. These alterations can influence membrane structure, intracellular signaling pathways, and macrophage cellular metabolism, ultimately contributing to tumor-promoting immune dysfunction [8]. However, how lipid profiling changes in the ovarian tumor microenvironment can affect the tumor-promoting properties of macrophages is not well understood.

Triglycerides, a major class of glycerolipids, function primarily as energy storage molecules but are increasingly recognized for their role in immune cell metabolism [9]. Accumulation of triglycerides within macrophages has been associated with enhanced lipid droplet formation, metabolic flexibility, and the adoption of immunosuppressive phenotypes [10]. Additionally, alterations in fatty acid chain length and saturation within triglyceride species may influence lipid signaling pathways, membrane properties, and macrophage polarization [11,12]. Despite these emerging insights, the temporal dynamics of triglyceride remodeling in TAMs and its relationship with global lipidomic changes during ovarian cancer progression have not been fully characterized.

In this study, we performed an exploratory LC-MS-based lipidomic analysis to characterize stage-associated differences in the lipid composition of CD11b^+^/F4/80^+^ macrophages isolated from ID8 tumor-bearing mice at Days 10, 40, and 80 following tumor cell inoculation. Our findings revealed stage-associated alterations in triglyceride species and lipid metabolism, providing a basis for future studies investigating whether altered macrophage lipid metabolism contributes an immunosuppressive/pro-tumoral macrophage state.

## 2. Materials and Methods

### 2.1. Isolation of Peritoneal Macrophages from Ovarian Tumor-Bearing Mice

All animal experiments were conducted in accordance with protocols approved by the Albany Medical College’s Institutional Animal Care and Use Committee (protocol number: 24-06001). Female C57BL/6 mice, 6–8 weeks of age, were purchased from Taconic Biosciences (Germantown, NY, USA) and maintained under pathogen-free conditions. For tumor establishment, 5 × 10^6^ ID8 ovarian cancer cells (Sigma, St. Louis, MO, USA) were suspended in serum-free DMEM (Gibco Life Technologies, Waltham, MA, USA), mixed with Matrigel (BD Biosciences, San Jose, CA, USA) at a 1:1 ratio, and administered by intraperitoneal injection. At Days 10 and 40 following tumor cell inoculation, when prominent ascites had not yet developed, 3% sodium thioglycollate (Sigma-Aldrich, St. Louis, MO, USA) was injected intraperitoneally 3 days before macrophage collection to facilitate recruitment and recovery of sufficient numbers of peritoneal macrophages. At Day 80, upon tumor cell injection, when mice developed advanced tumors accompanied by prominent ascites, macrophages were collected directly from the ascitic fluid without prior thioglycollate injection.

Peritoneal lavage or ascitic fluid was collected, and cells were centrifuged at 2000 rpm for 5 min. Red blood cells were removed using ACK lysis solution (Lonza, Basel, Switzerland). CD11b^+^ cells were enriched using magnetic-activated cell sorting (MACS) and subsequently subjected to fluorescence-activated cell sorting (FACS) to select F4/80^+^ cells. Six mice were used to collect CD11b^+^/F4/80^+^ macrophages each time point (total 18 mice). Macrophages from three mice were allocated for LC-MS-based lipidomic analysis, and macrophages from the remaining three mice were used for qPCR analysis. Samples from individual mice were analyzed separately and were not pooled.

### 2.2. Cell Culture

RAW 264.7 macrophages (a generous gift from Efthymia I. Matthaiou) were cultured in RPMI-1640 medium (Gibco Life Technologies, Waltham, MA, USA) supplemented with 10% fetal bovine serum (FBS; Sigma-Aldrich, St. Louis, MO, USA) and 1% antibiotic–antimycotic solution (Gibco Life Technologies, Waltham, MA, USA) and were maintained mycoplasma-free. RAW 264.7 cells were used between passages 3 and 9. To induce triglyceride accumulation, RAW 264.7 cells were treated with 250 µM oleic acid (OA; Sigma-Aldrich, St. Louis, MO, USA) conjugated to bovine serum albumin (BSA; Sigma-Aldrich, St. Louis, MO, USA) at a molar ratio of 2:1 for 24 h, which improves oleic acid solubility and cellular uptake. BSA alone served as the vehicle control. Treatment with OA did not significantly alter cell viability compared to the BSA-treated control group.

### 2.3. BODIPY Staining

LipidTOX neutral lipid stain (ThermoFisher, Waltham, MA, USA; #H34475) was used to visualize neutral lipid/lipid droplet accumulation, and DAPI (ThermoFisher, Waltham, MA, USA; #62248) was used to counterstain nuclei. Images were acquired using an Olympus BX61 (Olympus, Tokyo, Japan) upright microscope equipped with a PCO.edge 4.2 scientific CMOS camera (Excelitas Technologies Corp., Pittsburgh, PA, USA) and analyzed using MetaMorph software (version 7.10.2; Molecular Devices, San Jose, CA, USA).

### 2.4. Real-Time qPCR

RT-qPCR was performed using a CFX96 Real-Time PCR Detection System (Bio-Rad, Hercules, CA, USA), as previously described [13]. A total of 1 µg of total RNA was reverse-transcribed into cDNA using the RevertAid First strand cDNA synthesis kit (Thermo Fisher, Waltham, MA, USA; #K1622). Subsequently, 50 ng of cDNA was amplified using mouse gene-specific primers (Table 1) and iQSYBR Green Supermix (Bio-Rad, Hercules, CA, USA; #1708880). Relative gene expression was calculated using the 2^−ΔΔCt^ method.

### 2.5. Lipid Extraction and LC-MS/MS Analysis

CD11b^+^/F4/80^+^ macrophages isolated from tumor-bearing mice were submitted to the University of North Carolina’s Department of Chemistry Mass Spectrometry Core Laboratory for LC-MS-based lipidomic analysis. Cell pellets were subjected to MTBE-based lipid extraction. Briefly, cells were suspended in 1 mL methyl tert-butyl ether (MTBE) and thoroughly vortexed before being transferred to Eppendorf tubes. Methanol (300 µL) containing internal standards was subsequently added, and the samples were agitated for 10 min. Following the addition of 200 µL water, the samples were centrifuged at 2000× *g* for 10 min to induce phase separation. The upper organic phase was carefully collected, evaporated to dryness, and reconstituted in 100 µL isopropyl alcohol (IPA) for LC-MS analysis. A deuterated lipid mixture (EquiSPLASH; Avanti Polar Lipids, Alabaster, AL, USA) was incorporated as the internal standard. EquiSPLASH was added to methanol at a concentration of 1.5 µg/mL before extraction.

Lipidomic measurements were acquired using a Q Exactive Plus mass spectrometer (Thermo Fisher Scientific, Waltham, MA, USA) interfaced with an ACQUITY H-Class LC system (Waters Corporation, Milford, MA, USA). Chromatographic separation was performed on a Waters BEH C18 column (100 × 2.1 mm, 2.1 µm). Mobile phase A consisted of 60:40 (*v*/*v*) acetonitrile/water, whereas mobile phase B consisted of 90:10 (*v*/*v*) IPA/acetonitrile. Both mobile phases were supplemented with 10 mM ammonium formate and 0.1% formic acid. The LC flow rate was maintained at 0.2 mL/min. The gradient began at 32% B and was increased to 40% B at 1 min, with this composition maintained through 1.5 min. The proportion of B was subsequently increased to 45% at 4 min, 50% at 5 min, 60% at 8 min, 70% at 11 min, and 80% at 14 min. The 80% B composition was maintained through 16 min, after which the system was returned to the initial 32% B condition and equilibrated for 4 min.

Mass spectrometric data were collected using electrospray ionization with alternating positive and negative ionization modes. Data-dependent acquisition was performed using a top-5 MS/MS fragmentation strategy.

### 2.6. Lipid Identification and Data Processing

Raw LC-MS/MS files were processed using LipidSearch 5 (Thermo Fisher Scientific, Waltham, MA, USA). Lipid species with identification confidence grades A, B, or C were retained, followed by manual curation of annotations. Retention-time alignment used a retention-time tolerance of 0.05 min and a retention-time correction tolerance of 0.5 min. Lipid species were assigned based on MS/MS fragmentation data using mass tolerances of 5 ppm for precursor ions and 8 ppm for product ions. No blank subtraction was performed. A pooled quality-control sample was not generated for this batch; instead, the EquiSplash internal standard mixture was injected at the beginning of the run and after every 10 samples to monitor instrument performance throughout data acquisition. Injection order was not randomized. As all samples for this study were acquired within a single continuous run, formal batch-effect correction was not performed. Lipid identification was performed separately for each sample, followed by alignment of the resulting identifications across the designated sample groups (OxPAPC = C, HF = S1, and Con = S2). Adduct annotation included [M + H]^+^ and [M − H]^−^ as the primary adducts, with [M + HCOO]^−^ and [M + Na]^+^ additionally considered for selected lipid classes. Signal intensities were normalized using the EquiSPLASH deuterated lipid standard mixture (Avanti Polar Lipids, Alabaster, AL, USA).

The resulting dataset contained 2557 lipid species measured across three biological replicates at each time point (Day 10, Day 40, and Day 80). Data processing, statistical analyses, and visualization were performed in R (version 4.5.2; R Foundation for Statistical Computing, Vienna, Austria). Processed lipidomic data were imported from Microsoft Excel using the *readxl* package. Values reported as “NP” (not present/not detected) were converted to missing values (NA) before downstream analyses. The *dplyr* package was used to calculate replicate-level summary statistics, including per-lipid group means. Lipid abundance values were transformed using log10(x + 1) to reduce variance heterogeneity and improve the suitability of the data for downstream statistical analyses. Raw lipidomic data is provided as Supplementary File S1.

### 2.7. Differential Abundance Analysis

Differential lipid abundance was evaluated across the three tumor stages using pairwise comparisons of Day 10 versus Day 40, Day 10 versus Day 80, and Day 40 versus Day 80. For each lipid species, fold changes were calculated from the corresponding replicate-level mean abundances after log transformation. Statistical significance was assessed using Welch’s two-sample *t*-test, with the analysis performed on the individual biological replicate values rather than on group means. Volcano plots were generated using the ggplot2 package. For each pairwise comparison, *p*-values were adjusted for multiple testing using the Benjamini–Hochberg (BH) procedure, and lipid species with an adjusted *p*-value (FDR) < 0.05 were considered statistically significant (Supplementary File S2).

### 2.8. Multivariate Analysis

Principal component analysis (PCA) was performed using log10-transformed abundance across all nine biological samples to visualize overall variation in the macrophage lipid profiles across the three time points.

Partial least squares discriminant analysis (PLS-DA) was performed using the *mixOmics* package to explore lipidomic patterns associated with the three tumor stages. Since only three biological replicates were available per time point, PLS-DA was used for hypothesis generation. The PLS-DA model was evaluated using leave-one-out cross-validation. Models containing one and two components were evaluated based on their cross-validated classification error rates, and the two-component model was selected based on the observed cross-validation performance. Model stability was further assessed by permutation testing using 100 random permutations of the time-point labels. The resulting classification performance and permutation-test results were used to assess whether the observed group discrimination was unlikely to arise from random group assignment.

Variable importance in projection (VIP) scores was extracted from the two-component PLS-DA model. Since only three replicates were available, VIP scores were used solely as a ranking metric to identify candidate lipid species for visualization and were not interpreted as independent measures of statistical significance.

### 2.9. Heatmap Visualization

Hierarchical clustering heatmaps were generated using the *pheatmap* package. Z-score normalization was done on lipid abundance values prior to clustering. Heatmaps were generated for the full dataset as well as for the top 50 most variable lipid species selected by absolute fold change (Day 80 vs. Day 10) and by variance across all time points for TG-specific analyses.

### 2.10. Lipid Class and Subclass Analysis

For each biological replicate, the total abundance of each lipid class was calculated by summing the abundance of all detected lipid species belonging to that class. These replicate-level values were visualized as box plots using *ggplot2*, with each individual point representing one biological replicate. The relative contribution of each lipid class to the total lipid abundance was calculated and displayed as pie charts.

For the Day 80 versus Day 10 comparison, lipid species with a positive log10 fold change were classified as increased, whereas those with a negative log10 fold change were classified as decreased. The number and/or abundance contribution of each lipid class within these increased- and decreased-abundance pools was calculated and visualized using horizontal bar plots. This directional classification was used for descriptive visualization and did not by itself indicate statistical significance.

### 2.11. Triglyceride (TG) Species Analysis

TG species were extracted by selecting lipid annotations beginning with “TG”. Carbon chain length was derived by summing total acyl carbon parsed from TG species annotations and plotted against mean log2 abundance across time points. The total acyl carbon number for each TG species was derived from the summed carbon chain lengths reported in the lipid annotation. These values were plotted against the mean log2-transformed abundance of the corresponding TG species across the three time points. TG species were further categorized by their total number of double bonds across all three acyl chains, using three categories: 0, 1, or ≥2 total double bonds. This classification describes the overall degree of unsaturation of each TG species and does not indicate whether an individual fatty-acyl chain is saturated, monounsaturated, or polyunsaturated.

The total abundance of TG species within each total-double-bond category was visualized using stacked bar plots. To assess lipid-class remodeling according to overall degree of unsaturation, the log2 fold change (Day 80 versus Day 10) was calculated for each combination of lipid class and total double-bond number and displayed as a heatmap using *ggplot2*.

### 2.12. Correlation Network Analysis

Correlations were calculated using individual biological replicate-level abundance values, with pairwise complete observations where applicable. Correlation matrices were visualized using the *corrplot* package.

For network construction, correlation coefficients with an absolute value below 0.85 were set to zero. The resulting correlation matrix was used to construct an undirected, weighted network graph using the *igraph* package. Edge weights were defined by the absolute correlation coefficient, with positive correlations represented in red and negative correlations in blue. Network positioning was determined using the Fruchterman–Reingold force-directed algorithm based on absolute edge weights. Nodes were colored by lipid class, and community structure was explored using the Louvain algorithm with absolute edge weights.

Because the correlation analysis was based on a small number of biological replicates, the resulting correlations and network structure were considered exploratory and were not interpreted as evidence of direct biochemical interactions or causal relationships among lipid species.

### 2.13. KEGG Pathway Enrichment Analysis

Lipid species upregulated at each pairwise comparison (Day 40 vs. Day 10, Day 80 vs. Day 10, Day 80 vs. Day 40) were mapped to KEGG metabolic pathways based on lipid class annotations using a manually curated class-to-pathway reference table. The percentage of upregulated lipid species mapping to each pathway was calculated and visualized as horizontal bar plots using *ggplot2*.

### 2.14. Statistical Analysis

Statistical analyses were performed using GraphPad Prism version 7.0 (GraphPad Software, San Diego, CA, USA) and R version 4.5.2. Comparisons between two independent groups were performed using Welch’s two-sample *t*-test for lipidomics analysis, and Student’s *t*-test for qPCR analysis. For comparisons involving three or more groups, one-way ANOVA was used, followed by an appropriate post hoc multiple-comparison test when applicable. Statistical tests were performed using individual biological replicate-level observations, rather than group means. Data are presented as mean ± standard deviation (SD), with individual data points shown where applicable. *p*-value < 0.05 was considered statistically significant. For lipidomic analyses involving multiple comparisons, *p*-values were adjusted using the Benjamini–Hochberg procedure, and an adjusted *p*-value (FDR) < 0.05 was considered statistically significant.

## 3. Results

### 3.1. CD11b^+^/F4/80^+^ Macrophages Show Different Lipid Class Distribution at the Three Time Points Following ID8 Tumor Cell Injection

**Figure 1:**
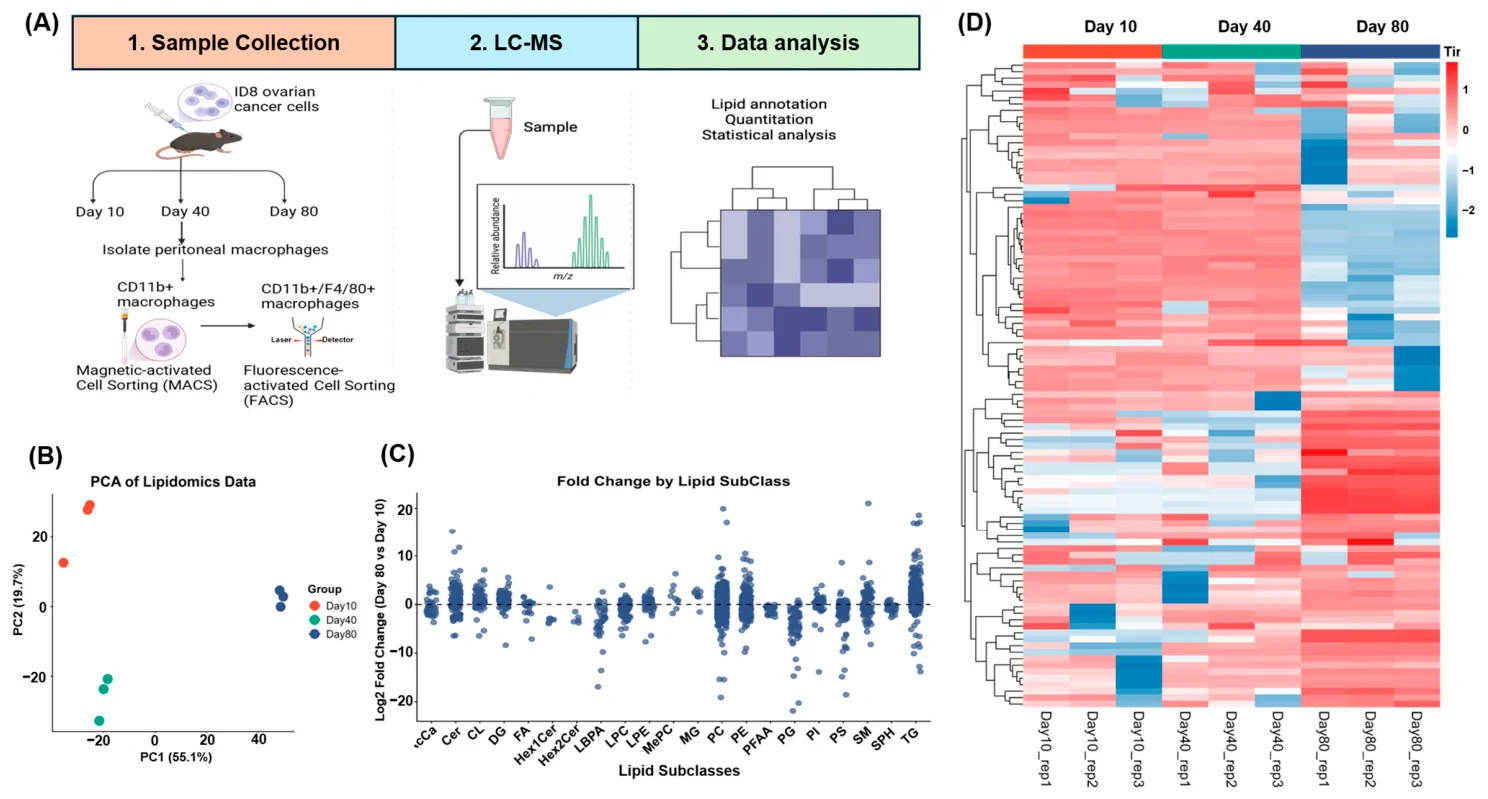
CD11b^+^/F4/80^+^ macrophages show different lipid class distribution at the three time points following ID8 tumor cell injection. (**A**) Schematic overview of the experimental design. (**B**) Principal component analysis (PCA) of lipidomic profiles from CD11b^+^/F4/80^+^ macrophages isolated at Days 10, 40, and 80 following ID8 tumor cell inoculation. Each dot represents one biological replicate (*n* = 3 per time point). (**C**) Dot plot showing fold changes in lipid class abundance among CD11b^+^/F4/80^+^ macrophages isolated at Days 10, 40 and 80. (**D**) Hierarchical clustering heatmap showing the distribution of detected lipid species across CD11b^+^/F4/80^+^ macrophage samples from the three time points.

To investigate the metabolic changes in macrophages at different time points after inducing ovarian cancer, we used a syngeneic ovarian cancer mouse model in which ID8 mouse ovarian cancer cells were injected intraperitoneally to female C57/BL6 mice. CD11b^+^/F4/80^+^ macrophages were isolated from tumor-bearing mice at Days 10, 40, and 80 following tumor cell injection. At Days 10 and 40, when prominent ascites had not developed, peritoneal macrophages were recovered following sodium thioglycollate administration, whereas at Day 80, macrophages were collected directly from tumor-associated ascitic fluid. CD11b^+^/F4/80^+^ macrophages were then subjected to LC-MS-based lipidomic analysis (Figure 1A, schematic).

Principal component analysis (PCA) was used to visualize overall variation in the macrophage lipidomic profiles across the three time points and revealed differences in macrophage global lipid profiles at Days 10, 40 and 80 (Figure 1B). Consistent with these observations, dot plot graph and hierarchical clustering confirmed differences in the distribution of lipid species among macrophages isolated at Days 10, 40, and 80 (Figure 1C,D).

**Figure 2:**
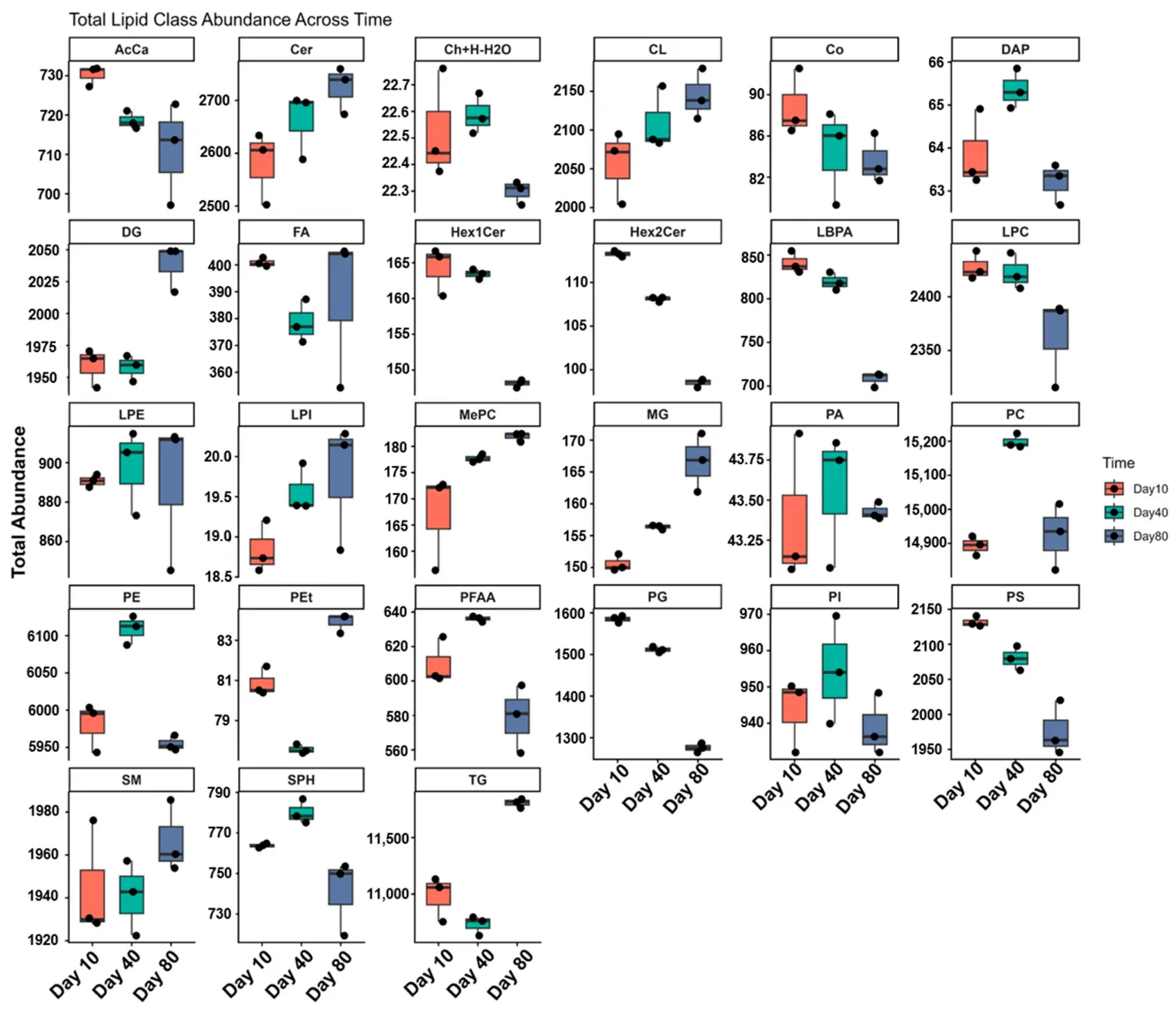
Total abundance of individual lipids in Cd11b^+^/F4/80^+^ macrophages isolated at different time points: box plot showing the total abundance of individual lipid classes in CD11b^+^/F4/80^+^ macrophages isolated at Days 10, 40, and 80 following ID8 tumor cell injection. Each point represents one biological replicate (*n* = 3 per time point).

Analysis of lipid classes revealed differences in lipid abundance at Day 80 relative to earlier time points. Several lipid classes, including ceramides (Cer), cardiolipins (CL), lysophosphatidylinositols (LPI), methyl-phosphatidylcholines (MePC), plasmalogen-phosphatidylethanolamines (PEt), sphingomyelins (SM), monoglycerides (MG), diglycerides (DG), and triglycerides (TG), showed increased abundance at Day 80 (Figure 1C and Figure 2). In contrast, several lipid classes showed lower abundance at Day 80 compared with earlier time points, including acylcarnitines (AcCa), diacylglycerol phosphate (DAP), hexosylceramides (Hex1Cer), dihexosylceramides (Hex2Cer), lysobisphosphatidic acid (LBPA), lysophosphatidylcholines (LPC), phosphatidylcholines (PC), phosphatidylethanolamines (PE), phosphatidylglycerols (PG), and phosphatidylserines (PS) (Figure 2).

Collectively, these findings indicate stage-associated differences in macrophage lipid composition in the ID8 ovarian cancer mouse model, including increased abundance of TG and other neutral lipid-related species at the late tumor stage.

### 3.2. CD11b^+^/F4/80^+^ Macrophages Showed Increased Abundance of TG Species at Day 80

**Figure 3:**
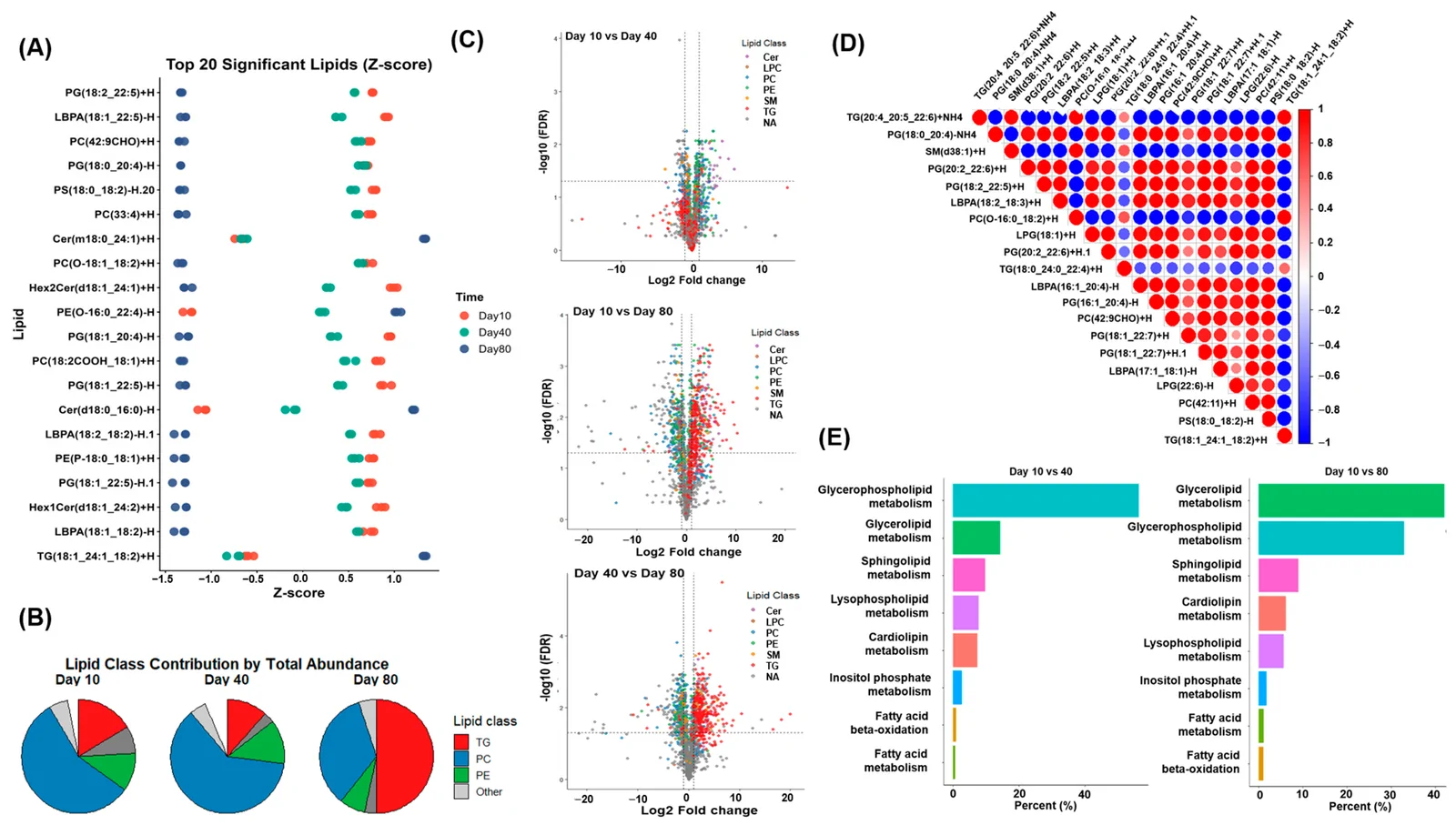
CD11b^+^/F4/80^+^ macrophages showed increased abundance of TG species at Day 80. (**A**) z-score dot plot showing the 20 most highly altered lipid species in CD11b^+^/F4/80^+^ macrophages isolated at Days 10, 40 and 80 following ID8 tumor cell injection. Each dot represents the z-score normalized abundance for an individual biological replicate. (**B**) Pie charts showing the relative contribution of major lipid classes, including triglycerides (TG), phosphatidylcholines (PC), and phosphatidylethanolamines (PE), to total measured lipid abundance at Days 10, 40, and 80. (**C**) Volcano plots showing differential lipid abundance between Days 10 and 40, Days 10 and 80 and Days 40 and 80. Each dot represents an individual lipid species. (**D**) Pairwise Pearson correlation matrix showing correlation among different lipid species in macrophages at Day 80. (**E**) KEGG pathway enrichment analysis showing the most enriched lipid metabolic pathways in macrophages for the pairwise comparisons.

To further characterize changes in lipid class distribution in CD11b^+^/F4/80^+^ macrophages across the three time points, we performed Z-score analysis and identified the 20 most highly altered lipid species based on their abundance patterns (Figure 3A). Several phospholipid species, including PC, PE, PS, and PG, showed higher abundance in macrophages from Days 10 and 40 and lower abundance at Day 80. In contrast, several Cer and TG species showed higher abundance at Day 80 compared to Days 10 Day 40 (Figure 3A). The relative contribution of individual lipid classes to the total lipid abundance further highlighted these differences (Figure 3B). PC represented the largest proportion of the macrophage lipidome at Day 10, whereas TG accounted for a smaller fraction. At Day 40, PC and PE remained prominent lipid classes, while the contribution of TG was modest. At Day 80, TG represented the dominant lipid class, accounting for nearly 50% of the total lipid abundance (Figure 3B). Volcano plots of pairwise comparisons between Days 10 and 40, Days 10 and 80, and Days 40 and 80 further showed that multiple TG species (indicated by red dots) were significantly abundant in macrophages at Day 80 compared with earlier time points (Figure 3C). Together, these results identify increased TG abundance as a prominent feature of the macrophage lipid profile at the late tumor stage.

Next, we examined relationships among representative lipid species using pairwise Pearson correlation analysis. Several phospholipid species showed strong negative correlations with TG species (Figure 3D). We further performed KEGG pathway enrichment analysis to identify metabolic pathways associated with the lipid species detected at different time points. At Day 40, glycerophospholipid metabolism was the highly enriched pathway, followed by glycerolipid metabolism (Figure 3E). At Day 80, glycerolipid metabolism showed the strongest enrichment among the identified pathways (Figure 3E). These results demonstrate stage-associated alterations in glycerolipid and glycerophospholipid metabolism, as well as an increased abundance of TG species in CD11b^+^/F4/80^+^ macrophages at the late tumor stage.

### 3.3. Highly Unsaturated and Longer-Chain TG Species Are Abundant in CD11b^+^/F4/80^+^ Macrophages Isolated at Day 80

**Figure 4:**
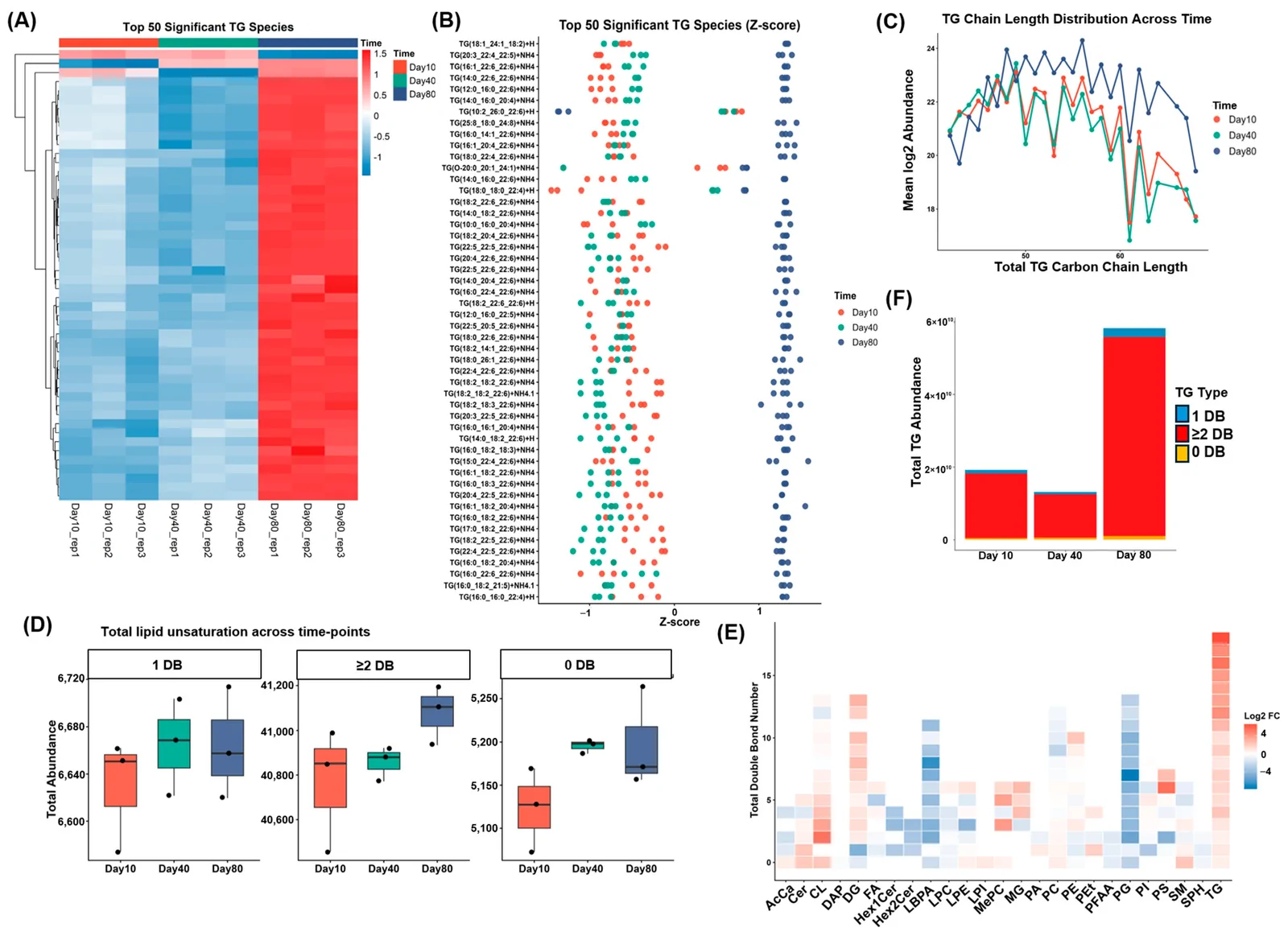
Highly unsaturated and longer-chain TG species are abundant in CD11b^+^/F4/80^+^ macrophages isolated at Day 80. (**A**) Heatmap showing hierarchical clustering of the top 50 most highly altered TG species across biological replicates of CD11b^+^/F4/80^+^ macrophages isolated at Days 10, 40, and 80 following ID8 tumor cell injection. Rows represent individual TG species (z-score normalized), and columns represent biological replicates. (**B**) Z-score dot plot showing the 50 most highly altered TG species across the three time points. Each dot represents the z-score normalized abundance of a TG species in an individual biological replicate. (**C**) Line graph showing mean log2 abundance of TG species categorized by total acyl carbon number at the three time points. (**D**) Box plots showing TG abundance categorized by total number of double bonds (DB; 0, 1, or ≥2) across Days 10, 40, and 80. Each dot represents one biological replicate (*n* = 3 per time point). (**E**) Tile heatmap depicting lipid class-by-unsaturation remodeling between Day 80 and Day 10. Each tile represents the log2 fold change for a given combination of lipid class (*x*-axis) and total double-bond number (*y*-axis). (**F**) Stacked bar plots showing the distribution of TG species according to total number of double bonds (0, 1, or ≥2) across the three time points.

Since TGs represented a prominent lipid class in CD11b^+^/F4/80^+^ macrophages at Day 80, we next analyzed which TG species were increased at Day 80. Heatmap and z-score distribution analyses showed increased abundance of multiple TG species at Day 80, with many of the top 50 TG species exhibiting higher abundance than at earlier time points (Figure 4A,B). To characterize compositional changes of TG species, we analyzed the distribution of total acyl carbon numbers among TG species across the three time points. TG species with longer total acyl carbon chains (>C50) were more abundant at Day 80 than at earlier time points (Figure 4C). We further assessed TG unsaturation based on the total number of double bonds across all three acyl chains and categorized TG species according to 0, 1, or ≥2 total double bonds. At Day 80, TG species with ≥2 total double bonds represented a substantial proportion of the TG species detected (Figure 4D), indicating an overall increase in the abundance of more highly unsaturated TG molecular species at the late tumor stage.

We next examined changes in total double-bond number across lipid classes at Day 80. Several lipid species, including CL, DG, MePC, MG, and TG, showed an increased distribution toward species with higher total numbers of double bonds at Day 80 (Figure 4E). In contrast, Hex1Cer, Hex2Cer, LBPA, and PG showed a distribution toward species with fewer total double bonds at Day 80 (Figure 4E). Importantly, nearly 90% of the TG species detected at Day 80 contained two or more total double bonds across their three acyl chains (Figure 4F). These findings demonstrate stage-associated differences in TG composition, characterized by increased abundance of TG species with longer-chain and higher total degrees of unsaturation in CD11b^+^/F4/80^+^ macrophages at the late tumor stage.

### 3.4. CD11b^+^/F4/80^+^ Macrophages at Day 80 Showed Increased Expression of Lipid Metabolism and Pro-Tumoral Macrophage-Associated Transcripts

**Figure 5:**
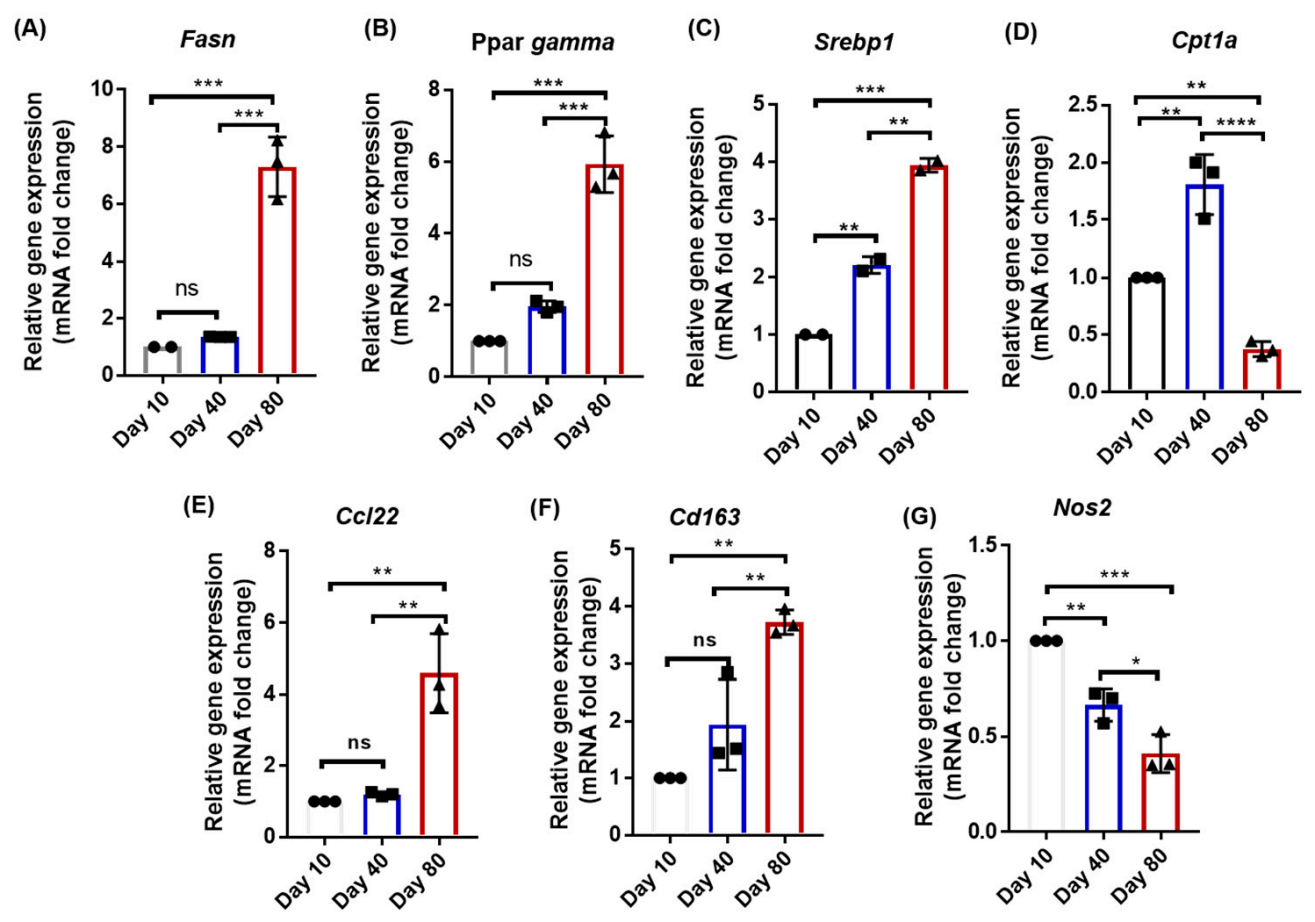
CD11b^+^/F4/80^+^ macrophages at Day 80 showed increased expression of lipid metabolism and pro-tumoral macrophage-associated transcripts. Bar graphs showing relative mRNA expression (fold change) of lipid metabolism genes (**A**) *Fasn*, (**B**) *Ppar-γ*, (**C**) *Srebp1*, and (**D**) *Cpt1a*, and transcripts associated with a pro-tumoral macrophage phenotype (**E**) *Ccl22*, (**F**) *Cd163*, and (**G**) *Nos2* in CD11b^+^F4/80^+^ macrophages isolated at Days 10, 40, and 80. Data are presented as mean ± SD (*n* = 3 per time point). Statistical significance was determined by one-way ANOVA followed by Tukey’s post hoc test. * *p* < 0.05, ** *p* < 0.01, *** *p* < 0.001, **** *p* < 0.0001; ns, not significant.

Since CD11b^+^/F4/80^+^ macrophages isolated at Day 80 exhibited increased abundance of TG species, we next examined whether these lipidomic differences were accompanied by changes in the expression of genes involved in lipid metabolism and macrophage functional state. For this, we isolated CD11b^+^/F4/80^+^ macrophages from ID8 tumor-bearing mice at Days 10, 40, and 80, and examined mRNA expression of genes associated with lipid metabolism and pro-tumoral macrophage states by RT-qPCR. CD11b^+^/F4/80^+^ macrophages isolated at Day 80 showed increased expression of *Fasn*, *Ppar gamma* and *Srebp1* compared to earlier time points (Figure 5A–C). We also observed decreased expression of *Cpt1a* in Day 80 macrophages (Figure 5D), implying a potential alteration in fatty acid utilization. Furthermore, we observed increased expression of *Ccl22* and *Cd163* and decreased expression of *Nos2* in macrophages at Day 80 compared to earlier time points (Figure 5E–G). Collectively, these findings support an association between altered macrophage lipid metabolism and pro-tumoral macrophage state at the late tumor stage.

### 3.5. Oleic Acid Treatment Increased Neutral Lipid Accumulation and Expression of Pro-Tumoral Phenotype-Associated Genes in RAW264.7 Macrophages

**Figure 6:**
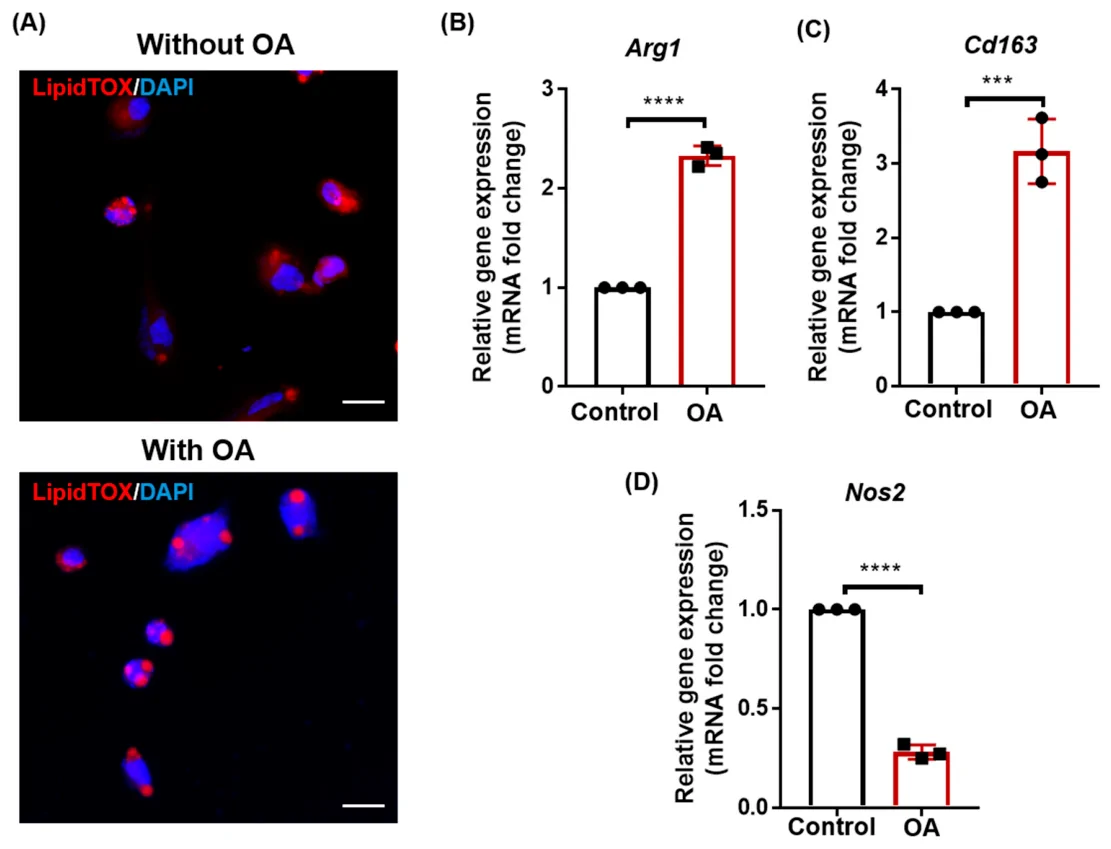
Oleic acid treatment increased neutral lipid accumulation and expression of pro-tumoral phenotype-associated genes in RAW264.7 macrophages. (**A**) Representative images of LipidTOX staining in RAW 264.7 macrophages treated with oleic acid (OA; 240 µM) or BSA control for 24 h. LipidTOX, red; DAPI, blue. Scale bar, 20 µm. (**B**–**D**) Bar graphs showing relative mRNA expression (fold change) of transcripts associated with a pro-tumoral macrophage phenotype (**B**) *Arg1*, (**C**) *Cd163*, (**D**) *Nos2* following OA treatment. Data are presented as mean ± SD (*n* = 3 per group). Statistical significance was determined by Student’s *t*-test. *** *p* < 0.001, **** *p* < 0.0001.

To further examine the relationship between neutral lipid accumulation and macrophage gene expression, we used exogenous oleic acid (OA) to increase neutral lipid accumulation in RAW 264.7 murine macrophages. RAW 264.7 macrophages were treated with OA (240 µM) for 24 h, and neutral lipid accumulation was assessed using LipidTOX neutral lipid stain. BSA-treated RAW 264.7 cells were used as controls. OA-treated macrophages exhibited increased LipidTOX staining compared to BSA-treated control macrophages, indicating accumulation of neutral lipids (Figure 6A). We next examined the expression of macrophage transcripts associated with a pro-tumoral phenotype by qPCR. OA treatment increased the expression of *Arg1* and *Cd163* compared to BSA-treated control macrophages (Figure 6B,C), whereas *Nos2* expression was decreased (Figure 6D). These results demonstrate that increased neutral lipid accumulation following OA treatment was accompanied by changes in macrophage gene expression associated with pro-tumoral macrophage state.

Collectively, our lipidomics and qPCR data provide evidence for an association between increased neutral lipid (particularly TG) accumulation and gene expression profile associated with pro-tumoral state in macrophages. Further mechanistic studies will be required to determine whether specific aspects of macrophage TG metabolism can regulate these phenotypic changes.

## 4. Discussion

Metabolic alterations are recognized as important features of the TME, enabling cancer cells to adapt to higher energy demand during disease progression. Lipids contribute to membrane structure, energy storage, signaling, and redox balance, and alterations in lipid metabolism have been reported in both cancer cells and cells within the TME [14,15,16,17,18,19,20]. Changes in macrophage metabolism have also been associated with alterations in macrophage function and phenotype. In the present study, we performed an exploratory lipidomic analysis of CD11b^+^/F4/80^+^ macrophages isolated from ID8 tumor-bearing C57/BL6 mice at Days 10, 40, and 80 following tumor cell injection. Our findings identified stage-associated alterations in macrophage lipid composition, lipid class distribution, and TG molecular species at different stages of ovarian cancer.

One of the most notable observations from this study was the increased abundance in TG species in CD11b^+^/F4/80^+^ macrophages isolated at Day 80. TGs accounted for nearly half of the total lipid abundance at this time point, consistent with increased neutral lipid storage in macrophages at the advanced tumor stage. Lipid droplet (LD) accumulation has been reported in macrophages in various cancers and has been associated with pro-tumorigenic macrophage functions [16,21]. Studies in other cancer models have also suggested that lipid-loaded macrophages can influence therapeutic responses and tumor progression [22]. For example, Wu et al. showed that accumulation of unsaturated fatty acids in macrophages can increase mitochondrial respiration and mTOR signaling, accompanied by increased expression of Cd206 and Arg1 [23]. Consistent with these observations, we found increased neutral lipid accumulation in RAW 264.7 macrophages following OA treatment.

Lipid droplets function as intracellular reservoirs of neutral lipids and can participate in fatty acid storage and mobilization, membrane lipid homeostasis, and cellular signaling [23]. The increased TG abundance and neutral lipid accumulation observed in Day 80 macrophages may therefore reflect changes in lipid storage and utilization in advanced stages of tumor development. However, our experimental design does not establish whether these changes represent metabolic adaptation within a stable macrophage population or differences in the composition of macrophage populations present at different stages of ovarian cancer. In addition, the different macrophage isolation procedures used at early and late stages represent an important potential confounding factor, as discussed below.

In addition to increased TG abundance, we observed enrichment of longer-chain TG species (≥C50) and TG species containing ≥2 total double bonds in CD11b^+^/F4/80^+^ macrophages isolated at Day 80. These findings indicate changes in the molecular composition of the TG pool at different stages of ovarian cancer. Fatty acid elongation and desaturation can influence membrane fluidity, lipid signaling, and metabolic flexibility [24]. Thus, increased levels of lipids with higher unsaturation may contribute to the regulation of oxidative stress responses and inflammatory signaling [25]. However, the presence of specific polyunsaturated fatty acids cannot be inferred solely from the total number of double bonds since the lipidomic measurements used in this study do not resolve the individual fatty-acyl chains within each TG species.

Beyond TGs, several other lipid classes also exhibited stage-associated changes. Macrophages isolated at Day 10 showed relatively greater abundance of PC and PG, core structural membrane components [26], whereas TG abundance increased at Days 40 and 80. These findings are consistent with a shift from membrane-associated pools toward lipid storage pools during tumor progression [27]. Because lipid metabolism is closely connected to macrophage activation and function, such alterations may be relevant to macrophage adaptation within the TME [28,29,30]. However, the present study does not establish whether the observed lipid changes directly regulate macrophage function or tumor progression.

Correlation network analysis further demonstrated relationships among multiple lipid species, including inverse correlations between several phospholipid and TG species. These correlations may reflect coordinated changes in lipid metabolism and broader metabolic network adaptations. However, correlation does not establish direct biochemical interactions or causal relationships among lipid species. Pathway enrichment analysis also revealed prominent enrichment of glycerolipid metabolism at Day 80, consistent with the increased abundance of TGs at this stage. These findings corroborate previous studies showing pro-tumorigenic features in lipid-laden macrophages [31,32]. Additional pathways, including sphingolipid and cardiolipin metabolism, also showed enrichment at Day 80, suggesting that mitochondrial function, membrane dynamics, and signaling processes may contribute to the metabolic adaptations observed in ovarian cancer progression [33,34,35,36]. However, these findings provide an exploratory and descriptive view of lipid metabolic remodeling associated with different stages of tumor development but do not establish that glycerolipid metabolism is a driver of macrophage phenotypic changes.

Our gene expression analyses provide additional evidence of stage-associated changes in macrophage lipid metabolism and phenotype-associated transcripts. Increased expression of *Fasn*, *Pparγ*, and *Srebp1*, together with decreased *Cpt1a*, reflects changes in lipid synthesis, storage, and fatty acid utilization, and is consistent with altered lipid metabolic pathways in Day 80 macrophages. We also observed increased expression of *Ccl22* and *Cd163* and decreased expression of *Nos2* at Day 80, representing a gene expression profile associated with a pro-tumoral macrophage phenotype. Similarly, OA treatment increased neutral lipid accumulation, accompanied by changes in these transcripts in RAW 264.7 macrophages. These findings support an association between altered macrophage lipid metabolism and a pro-tumoral macrophage phenotype. However, these findings do not establish that altered macrophage lipid metabolism and increased *de novo* lipogenesis or TG accumulation itself causes a pro-tumoral macrophage phenotype.

Several limitations should be considered when interpreting these findings. First, macrophages were isolated using two different procedures across time points. Sodium thioglycollate was used to recruit and enrich peritoneal macrophages at Days 10 and 40 when ascites had not yet developed, whereas macrophages were collected directly from ascitic fluid at Day 80. Because thioglycollate is an inflammatory stimulus that can influence macrophage recruitment and activation, isolation procedure and tumor stage were confounded in this design. Second, CD11b^+^/F4/80^+^ selection enriches macrophages but does not fully resolve macrophage heterogeneity or establish a specific functional state. Third, this study did not include an age-matched tumor-free control group. Day 10 represents an early-stage tumor-bearing reference time point, rather than a healthy control. Fourth, the study used a cross-sectional time-point comparison rather than longitudinal analysis of the same animals or macrophage populations. Thus, the observed differences cannot distinguish metabolic changes within a stable macrophage population from changes in macrophage composition or replacement by distinct macrophage subsets during tumor progression. Finally, the lipidomic analysis included only three biological replicates per time point, which limits statistical power and the generalizability of the findings. Although the PLS-DA model was evaluated by cross-validation and permutation testing, the small number of biological replicates limits the robustness of model-based classification and feature ranking. Thus, these results should be interpreted as descriptive and hypothesis-generating rather than as confirmatory evidence of reproducible group discrimination.

Future studies using matched macrophage isolation procedures, age-matched tumor-free controls, larger sample sizes, and more comprehensive macrophage phenotyping will help distinguish tumor-associated metabolic alterations from effects related to the sampling procedure or macrophage population composition. Additionally, it will be necessary to determine the functional consequences of TG accumulation in macrophage phenotype and to identify the specific enzymes or regulatory pathways responsible for these alterations in macrophages in ovarian cancer.

Given the importance of altered lipid metabolism in ovarian cancer progression, understanding how macrophage lipid metabolism interacts with cancer cells and other components of the TME may help elucidate mechanisms underlying disease progression and develop new therapeutic strategies for ovarian cancer.

## 5. Conclusions

Collectively, our findings identified stage-associated alterations in the lipidomic profiles of CD11b^+^/F4/80^+^ macrophages from ID8 tumor-bearing mice, including increased TG abundance. These findings are accompanied by changes in the expression of genes involved in lipid synthesis and storage as well as a gene expression profile associated with a pro-tumoral macrophage state. These results provide a foundation for future studies to determine whether macrophage lipid metabolism contributes to a pro-tumoral macrophage state and ovarian cancer progression.

## Figures and Tables

**Table 1:** Mouse gene forward and reverse primer sequences used in this study.

Gene Name	Forward Primer (5′–3′)	Reverse Primer (5′–3′)
*Arg1*	CATTGGCTTGCGAGACGTAGAC	GCTGAAGGTCTCTTCCATCACC
*Cd163*	GGCTAGACGAAGTCATCTGCAC	CTTCGTTGGTCAGCCTCAGAGA
*Nos2*	GAGACAGGGAAGTCTGAAGCAC	CCAGCAGTAGTTGCTCCTCTTC
*Fasn*	CACAGTGCTCAAAGGACATGCC	CACCAGGTGTAGTGCCTTCCTC
*Ppar-γ*	GTACTGTCGGTTTCAGAAGTGCC	ATCTCCGCCAACAGCTTCTCCT
*Srebp1*	CGGAAGCTGTCGGGGTAG	GTTGTTGATGAGCTGGAGCA
*Cpt1a*	GGCATAAACGCAGAGCATTCCTG	CAGTGTCCATCCTCTGAGTAGC
*Ccl22*	GTGGAAGACAGTATCTGCTGCC	AGGCTTGCGGCAGGATTTTGAG

## Data Availability

The original contributions presented in this study are included in the article/Supplementary Material. Further inquiries can be directed to the corresponding author.

## Supplementary Materials

The following supporting information can be downloaded at https://www.mdpi.com/article/10.3390/cancers18182959/s1, Supplementary File S1: Raw lipidomic data; Supplementary File S2: Adjusted *p*-values across lipid species at different time points.

## Abbreviations

The following abbreviations are used in this manuscript:

Cer	Ceramide
CL	Cardiolipin
LPI	Lysophosphatidylinositol
MePC	Methyl-phosphatidylcholine
PEt	Plasmalogen-phosphatidylethanolamine
SM	Sphingomyelin
TG	Triglyceride
MG	Monglyceride
DG	Diglyceride
AcCa	Acylcarnitine
DAP	Diacylglycerol phosphate
Hex1Cer	Hexosylceramide
Hex2Cer	Dihexosylceramide
LBPA	Lysobisphosphatidic acid
LPC	Lysophosphatidylcholine
PC	Phosphatidylcholine
PE	Phosphatidylethanolamine
PFAA	Primary fatty acid amide
PG	Phosphatidylglycerol
PS	Phosphatidylserine
SFA	Saturated fatty acid
MUFA	Monounsaturated fatty acid
PUFA	Polyunsaturated fatty acid
OA	Oleic acid
FASN	Fatty acid synthase
PPAR-γ	Peroxisome proliferator-activator receptor-gamma
SREBP	Sterol regulatory element-binding protein
CPT1A	Carnitine palmitoyltransferase 1A
MTBE	Methyl tert-butyl ether
TME	Tumor microenvironment
TAM	Tumor-associated macrophage
LD	Lipid droplet
